# Hydrocortisone and Risk Factors for Kidney Replacement Therapy in Septic Shock

**DOI:** 10.1001/jamanetworkopen.2025.12279

**Published:** 2025-05-27

**Authors:** Lachlan H. Donaldson, Anthony Devaux, Kyle C. White, Dorrilyn Rajbhandari, Jeremy Cohen, Rinaldo Bellomo, John Myburgh, Naomi Hammond, Balasubramanian Venkatesh

**Affiliations:** 1 The George Institute for Global Health, Faculty of Medicine, University of New South Wales, Sydney, Australia; 2 Malcolm Fisher Department of Intensive Care Medicine, Royal North Shore Hospital, St Leonards, Australia; 3 Northern Clinical School, Sydney Medical School, University of Sydney, Sydney, Australia; 4 Princess Alexandra and Wesley Hospitals, Brisbane, Australia; 5 School of Clinical Sciences, Faculty of Health, Queensland University of Technology, Brisbane, Australia; 6 The Wesley Hospital, Brisbane, Australia; 7 Faculty of Medicine, University of Queensland, Brisbane, Australia; 8 Department of Intensive Care, Austin Hospital, Melbourne, Australia; 9 Australian and New Zealand Intensive Care Research Centre, Monash University, Melbourne, Australia; 10 Department of Critical Care, The University of Melbourne, Melbourne, Australia; 11 Department of Intensive Care Medicine, St George Hospital, Kogarah, Australia; 12 Department of Intensive Care Medicine, Gold Coast University Hospital, Southport, Australia.

## Abstract

**Importance:**

Sepsis-associated acute kidney injury (SA-AKI) is a common and clinically important condition in patients who are critically ill. Dysregulated inflammation may contribute to it. Intravenous hydrocortisone may decrease the risk of SA-AKI progression.

**Objective:**

To describe the associations of hydrocortisone use with the incidence and outcomes of requirement for kidney replacement therapy (KRT), as well as source of sepsis, mean arterial pressure (MAP), and MAP indexed to required vasopressor (norepinephrine equivalent [NEE]).

**Design, Setting, and Participants:**

This cohort study was conducted as a post hoc analysis of the Adjunctive Corticosteroid Treatment in Critically Ill Patients with Septic Shock (ADRENAL) randomized clinical trial (RCT), a multicenter placebo-controlled RCT of hydrocortisone in patients with septic shock in 69 intensive care units in Australia, the United Kingdom, New Zealand, Saudi Arabia, and Denmark that recruited between 2013 and 2017. Participants were patients enrolled in the ADRENAL study with septic shock who did not require KRT in the 24 hours prior to randomization and who did not have a prior longstanding dialysis requirement. Data were analyzed between July and September 2024.

**Exposures:**

Receipt of hydrocortisone (vs placebo), MAP at enrollment, vasopressor dose (NEE) and MAP:NEE ratio, source of sepsis, causative organism, bacteremia, and the use of nephrotoxic antimicrobials, vasopressin, or specific inotropes.

**Main Outcomes and Measures:**

Outcomes of interest were KRT requirement and liberation from KRT, measured as days alive and free of KRT.

**Results:**

A cohort of 3161 patients (median [IQR] age, 65 [53-74] years, 1921 [61%] male) was identified, including 1589 patients randomized to receive hydrocortisone and 1572 patients who received the placebo. Allocation to treatment with hydrocortisone was associated with a significantly reduced incidence of KRT requirement compared with placebo (329 patients [21%] vs 372 patients [24%]; odds ratio [OR], 0.84 [95% CI, 0.70 to 0.99]; *P* = .04). When controlled for factors associated with KRT requirement, randomization to hydrocortisone remained significantly associated with a reduced odds of new KRT requirement (OR, 0.79 [95% CI, 0.66 to 0.95]; *P* = .01). Among patients who started KRT following randomization, hydrocortisone was not associated with reduced days alive and free of KRT (mean difference, 1.28 [95% CI, −4.31 to 6.87] days; *P* = .65).

**Conclusions and Relevance:**

In this post hoc cohort study of patients with septic shock enrolled in a large RCT, intravenous hydrocortisone was associated with a reduced risk of new KRT requirement following randomization.

## Key Points

**Question:**

In patients with septic shock, is the administration of intravenous hydrocortisone independently associated with subsequent rates of kidney replacement therapy (KRT)?

**Findings:**

In this cohort study using a post hoc analysis of 3161 patients enrolled to a randomized clinical trial of hydrocortisone in severe septic shock who had not yet been initiated on KRT, treatment with hydrocortisone was associated with significantly reduced odds of new KRT requirement. This association was robust to adjustment for other risk factors for KRT requirement.

**Meaning:**

The findings of this study suggest that use of intravenous hydrocortisone in septic shock warrants further investigation.

## Teaser

This cohort study examines the associations of hydrocortisone use with the incidence and outcomes of requirement for kidney replacement therapy (KRT) for patients with septic shock.

## Introduction

The incidence of sepsis-associated acute kidney injury (SA-AKI) among patients admitted to the intensive care unit (ICU) has been reported to have increased between 2015 and 2021 and now occurs in up to 18% of all patients admitted to the ICU.^1^ Among ICU patients with sepsis specifically, the incidence of AKI approaches 52% and carries a high risk of death and persisting kidney impairment among survivors.^2^

The pathophysiological mechanisms underlying SA-AKI are driven by dysregulated inflammatory responses in the kidney leading to changes in microvascular perfusion, local inflammatory toxic effects, and cellular metabolic changes prioritizing cellular survival over function.^3^ SA-AKI appears not to be characterized by macrovascular hypoperfusion and associated ischemia.^4,5^

Given SA-AKI is driven primarily by dysregulated inflammatory response, corticosteroids may have a role in tempering that inflammation. Hydrocortisone has been extensively researched as an adjunctive therapy in septic shock.^6^ Explorations of the impact of hydrocortisone on rates of SA-AKI have been limited in most large recent trials by a high proportion of patients with advanced AKI, or established dialysis dependence, at the time of study enrollment.^7,8,9^ A number of smaller trials with low rates of advanced AKI at enrollment have not demonstrated a difference in subsequent kidney replacement therapy (KRT) requirement.^10,11^

This study was a post hoc analysis of a subgroup of patients enrolled into a multinational randomized clinical trial (RCT), the Adjunctive Corticosteroid Treatment in Critically Ill Patients with Septic Shock (ADRENAL) trial. The ADRENAL trial was an RCT of intravenous hydrocortisone 200 mg or placebo daily for 7 days in patients who were critically ill with septic shock and receiving mechanical ventilation. The primary outcome of the study was 90-day mortality, and secondary outcomes included days alive and free of KRT and incident KRT requirement. Further exploration of the impact of hydrocortisone on kidney outcomes in the ADRENAL study was limited by high rates of established severe AKI at enrollment (13% receiving KRT) and the lack of dedicated analyses controlling for risk factors for KRT requirement.

The primary aim of this post hoc analysis was to describe the association of hydrocortisone use with the incidence and outcomes of subsequent KRT requirement, when controlling for other factors found to be associated with KRT requirement, including differing sources of sepsis and measures of hemodynamic compromise. Secondary aims were to describe other associations with KRT requirement and to describe the long-term outcomes associated with an acute KRT requirement in septic shock, including measures of health-related quality of life (HRQOL).

## Methods

### Setting and Participants

The ADRENAL trial recruited 3800 patients from Australia, the United Kingdom, New Zealand, Saudi Arabia, and Denmark between 2013 and 2017. Recruited patients were admitted to the ICU with suspected sepsis, were mechanically ventilated, and required vasopressors or inotropes.^7^ Outcomes were reported up to 6 months following randomization. The study was approved by a human research ethics committee at all study sites prior to patient enrollment. All participants provided informed consent. This study is reported following the Strengthening the Reporting of Observational Studies in Epidemiology (STROBE) reporting guideline.

This post hoc analysis includes all patients enrolled in the ADRENAL study who did not require KRT in the 24 hours prior to randomization and who did not report a dialysis requirement for chronic kidney disease in the 12 months prior to randomization. Due to a low proportion of missing data, all analyses were performed using complete cases. We excluded 153 patients (4.8%) from the multivariate analysis, mostly due to the absence of bilirubin measurement at baseline.

### Exposures

Country of enrollment, admission source and type (operative or nonoperative), and treatment allocation (hydrocortisone or placebo) were described, along with other factors associated with outcome at enrollment: age, sex, weight, Acute Physiology and Chronic Health Evaluation (APACHE) II score,^12^ and liver dysfunction. The primary source of sepsis was described, as well as the first organism identified and the presence of bacteremia. The use of nephrotoxic antibiotics was recorded, including amikacin, amphotericin B, gentamicin, polymyxin B, tobramycin, or vancomycin.

The degree of hemodynamic instability at study enrollment was described as the required norepinephrine equivalent (NEE) at study enrollment, which was calculated based on the norepinephrine, adrenaline, and dopamine doses at a single time point at study enrollment, with adjustment if the patient was noted to be receiving vasopressin^13^ (eAppendix in Supplement 1). The mean arterial pressure (MAP) indexed to NEE (MAP:NEE ratio) was calculated.^14^ The use of vasopressin at the time of enrollment was described, as well as the use of specific inotropes (dobutamine, milrinone, or levosimendan).

### Outcomes

The primary outcome for the study was the incidence of new KRT requirement following randomization. KRT requirement was used as a surrogate for severe SA-AKI, acknowledging KRT may occasionally be initiated for alternative indications, such as fluid overload or severe acidemia. For patients who initiated KRT, the number of days alive and free of KRT to day 90 was described for successful liberation from KRT to account for death as a competing risk.

Other outcomes reported for patients who did and did not require KRT included days alive and out of the ICU and hospital, mortality, and HRQOL. HRQOL was measured at 6 months, as previously reported.^15^ Briefly, at 6 months after randomization, patients were assessed using the EQ-5D-5L^16^ by blinded, trained staff. HRQOL utility values were calculated using the Australian algorithm with values generally ranging between less than 0 (worse than death), through to 0 (death) and 1 (perfect health).^17^ In addition, using the visual analogue scale (VAS), respondents were asked to rate their perceived health on a scale of 0 (worst) to 100 (best). Postrandomization variables were also described, including time to shock resolution and rates of enduring shock resolution, defined by a recurrent requirement for vasopressors after successful weaning.

### Statistical Analysis

Data are presented as simple counts or proportions, or as means with SDs, or medians with IQRs, as appropriate. The primary analysis compared incident KRT requirement and days alive and free of KRT between patients randomized to hydrocortisone vs placebo (analyzed by intention-to-treat), with adjustment for admission type as a fixed effect, and study sites as a random effect, as was the analysis of KRT requirement in the ADRENAL trial.^7^ The association of epidemiological and shock-related factors with subsequent KRT use was further explored by comparing the incidence of these risk factors between patients who did and did not require KRT. The log of the odds of the KRT requirement probability was analyzed using logistic regression. The exposure associations were presented using odds ratios (ORs) and 95% CIs. The multivariate model included all exposures with a statistically significant association on the univariate analysis (*P* < .05), as well as those defined for inclusion a priori: age, sex, APACHE II, bilirubin at enrollment, primary site of infection, time from the onset of shock to randomization, use of vasopressin, last MAP at enrollment, and ADRENAL treatment allocation.

Further sensitivity analyses were performed that compared incident KRT requirement between treatment groups. First, analyses were performed with further adjustment for age, sex, APACHE II score, bilirubin at enrollment, primary site of infection, time from the onset of shock to randomization, use of vasopressin and last MAP at enrollment as fixed effects. Second, we repeated analyses excluding patients who developed a KRT requirement within 1 and 2 days of randomization.

For analysis of outcomes among patients who developed a KRT requirement, for each exposure, the mean difference in days alive and free of KRT was also described in both a univariate and multivariate analyses. For analysis of the HRQOL data, the mean 6-month utility score was compared using the *t* test. For the primary analysis, deceased patients were coded a score of 0; a sensitivity analysis that included only survivors was included in eTable 1 in Supplement 1. The mean EQ-5D-5L VAS was also compared between groups using the *t* test; only survivors were included in this analysis.

Statistical significance for all analyses was determined using a 2-sided hypothesis test with α = .05. Analyses were performed using R software version 4.4.2 (R Project for Statistical Computing). Statistical analyses for this post hoc study were performed from July to September 2024.

## Results

**Figure 1.**
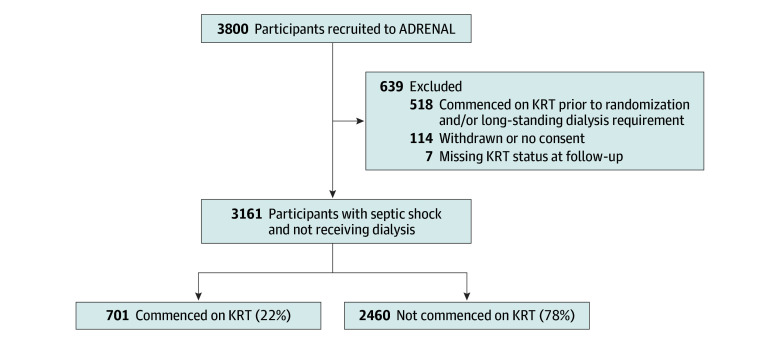
Selection and Outcome of Participants in Cohort ADRENAL indicates Adjunctive Corticosteroid Treatment in Critically Ill Patients with Septic Shock; KRT, kidney replacement therapy.

After exclusions (Figure 1), a cohort of 3161 patients (median [IQR] age, 65 [53-74] years, 1921 [61%] male) was identified, including 1589 patients randomized to receive hydrocortisone and 1572 patients who received the placebo (Table 1). Most participants were recruited from Australia and New Zealand (2628 patients [72%]).

**Table 1.** Baseline Patient Characteristics by KRT Status

Variables	Patients, No./total No. (%)	
	KRT (n = 701)	No KRT (n = 2460)
Age at randomization, mean (SD), y	62.3 (14.3)	62.4 (15.5)
Sex		
Female	265/701 (38)	975/2460 (40)
Male	436/701 (62)	1485/2460 (60)
Weight, mean (SD), kg	88.9 (27.5)	84.1 (25.9)
APACHE II score, mean (SD)	27.0 (7.3)	22.1 (7.2)
Highest bilirubin, median (IQR), mg/dL	1.2 (0.8-2.3)	1.0 (0.6-1.6)
Country		
Australia	494/701 (70)	1779/2460 (72)
Denmark	26/701 (4)	108/2460 (4)
New Zealand	79/701 (11)	276/2460 (11)
Saudi Arabia	26/701 (4)	87/2460 (4)
United Kingdom	76/701 (11)	210/2460 (9)
Source of admission		
Accident and emergency department	246/701 (35)	660/2460 (27)
Hospital floor (ie, wards)	171/701 (24)	560/2460 (23)
Transfer from another ICU	34/701 (5)	99/2460 (4)
Transfer from another hospital (except from another ICU)	76/701 (11)	233/2460 (10)
Admitted from emergency surgery	154/701 (22)	789/2460 (32)
Admitted from elective surgery	20/701 (3)	119/2460 (5)
Admission type: operative	169/701 (24)	893/2460 (36)
Septic source and antibiotic use		
Primary site of infection		
Pulmonary	232/698 (33)	898/2456 (37)
Intra-abdominal	121/698 (17)	562/2456 (23)
Blood	147/698 (21)	381/2456 (16)
Urinary	140/698 (20)	452/2456 (18)
Other	58/698 (8)	163/2456 (7)
First organism cultured		
* Staphylococcus*	40/698 (6)	129/2455 (5)
Other gram-positives	146/698 (21)	502/2455 (20)
Gram-negatives	159/698 (23)	469/2455 (19)
Fungi	40/698 (6)	120/2455 (5)
Other (including none identified)	313/698 (45)	1235/2455 (50)
Bacteremia present	142/701 (20)	300/2458 (12)
Use of nephrotoxic antibiotics at randomization^a^	325/701 (46)	1006/2460 (41)
Hemodynamic instability and management		
Last MAP, median (IQR), mm Hg	71.0 (67.0-76.0)	72.0 (68.0-77.5)
Lowest MAP in previous 24 h, median (IQR), mm Hg	57.0 (50.0-62.0)	59.0 (53.0-64.0)
NEE, median (IQR), µg/kg/min	0.26 (0.13-0.43)	0.15 (0.08-0.28)
MAP:NEE ratio, mean (SD)	538.4 (901.9)	820.1 (1137.5)
CVP at randomization, median (IQR)	13.0 (10.0-16.0)	11.0 (8.0-14.0)
Use of vasopressin	172/701 (25)	295/2460 (12)
Use of dobutamine, milrinone, or levosimendan	37/701 (5)	76/2460 (3)
Time from the onset of shock to randomization, mean (SD), h	22.9 (110.7)	19.6 (86.5)
Corticosteroid received prior to randomization	72/646 (11)	200/2264 (9)
ADRENAL treatment allocation		
Placebo	372/701 (53)	1200/2460 (49)
Hydrocortisone	329/701 (47)	1260/2460 (51)

Abbreviations: ADRENAL, Adjunctive Corticosteroid Treatment in Critically Ill Patients with Septic Shock; APACHE, Acute Physiology and Chronic Health Evaluation; CVP, central venous pressure; ICU, intensive care unit; KRT, kidney replacement therapy; MAP, mean arterial pressure; NEE, norepinephrine equivalent.

SI conversion factor: To convert bilirubin to micromoles per liter, multiply by 17.104.

^a^ Nephrotoxic antibiotics included amikacin, amphotericin B, gentamicin, polymyxin B, tobramycin, or vancomycin.

A total of 701 patients (22%) had new requirement of KRT following enrollment in ADRENAL. The median (IQR) time to initiation of KRT from study enrollment was 1 (0-3) days, and the median (IQR) duration of KRT requirement was 5 (3-11) days.

### Baseline Characteristics and Incident KRT Requirement

Pulmonary sepsis accounted for 36% of primary infections overall (1132 patients), followed by intra-abdominal infections (683 patients [22%]). Gram-positive organisms were the first organisms identified in 817 patients (26%), and gram-negative organisms were cultured in 628 patients (20%). A total of 442 patients (14%) had bacteremia. The median (IQR) APACHE II score was 23 (18-28), and patients who developed a subsequent KRT requirement had a higher APACHE II score compared with those who did not require KRT (mean [SD] score, 27.0 [7.3] vs 22.1 [25.9]).

At the time of study enrollment, the MAP between patients who did and did not subsequently require KRT was similar (median [IQR], 71.0 [67.0-76.0] mm Hg vs 72.0 [68.0-77.5] mm Hg). Patients who subsequently developed a KRT requirement had a higher vasopressor dose (mean [SD] NEE, 0.33 [0.29] µg/kg/min vs 0.22 [0.24] µg/kg/min) and higher vasopressor requirement for a given blood pressure (mean [SD] MAP:NEE ratio, 538.4 [901.9] mm Hg/µg/kg/min vs 820.1 [1137.5] mm Hg/µg/kg/min) (Table 1).

### Hydrocortisone and KRT Requirement

Allocation to treatment with hydrocortisone was associated with a significantly reduced incidence of KRT requirement (329 patients [21%]) compared with placebo (372 patients [24%]) (OR, 0.84 [95% CI, 0.70-0.99]; *P* = .04) (Table 2). In a multivariate model that included factors found to be associated with KRT requirement, randomization to hydrocortisone remained significantly associated with a reduced odds of KRT requirement (OR, 0.79 [95% CI, 0.66-0.95]; *P* = .01) (Table 3). Further sensitivity analyses demonstrated that the reduced incident requirement for KRT among patients allocated to hydrocortisone was robust to further adjustment for other risk factors for AKI (age, sex, APACHE II score, bilirubin at enrollment, primary site of infection, time from the onset of shock to randomization, use of vasopressin) (adjusted OR, 0.81 [95% CI, 0.68-0.97]; *P* = .02) (eTable 2 in Supplement 1). After exclusion of patients who commenced KRT early following treatment allocation (within 1-2 days following randomization), the incidence of KRT requirement remained lower in the hydrocortisone group (1 day: OR, 0.82 [95% CI, 0.66-1.02]; *P* = .08; 2 days: OR, 0.78 [95% CI, 0.59-1.01]; *P* = .06).

**Table 2.** New KRT Requirement Following Randomization Among Patients Allocated to Hydrocortisone vs Placebo

Outcomes	Treatment allocation		Estimate (95% CI)^a^	*P* value
	Hydrocortisone	Placebo		
Use of KRT, No./total No. (%)	329/1589 (21)	372/1572 (24)	0.84 (0.70 to 0.99)^b^	.04
Days alive and free of KRT, mean (SD)	69.6 (34.0)	68.1 (35.0)	1.54 (−0.86 to 3.93)^c^	.21

Abbreviation: KRT, kidney replacement therapy.

^a^ Adjusted for admission type as a fixed effect, and sites as a random effect.

^b^ Expressed as an odds ratio.

^c^ Expressed as a mean difference.

**Table 3.** Univariate and Multivariate Associations With the Development of a KRT Requirement

Variable	Outcome, No./total No. (%)		Univariate		Multivariate^a^		
	KRT (n = 701)	No KRT (n = 2460	OR (95% CI)	*P* value	OR (95% CI)	*P* value
Age at randomization, mean (SD) y	62.3 (14.3)	62.4 (15.5)	1.00 (0.99-1.01)	.87	1.00 (0.99-1.00)	.31
Sex						
Female	265/701 (38)	975/2460 (40)	1 [Reference]	.38	1 [Reference]	.98
Male	436/701 (62)	1485/2460 (60)	1.08 (0.91-1.28)		1.00 (0.82-1.21)	
Weight, mean (SD), kg	88.9 (27.5)	84.1 (25.9)	1.01 (1.00-1.01)	<.001	1.01 (1.01-1.01)	<.001
APACHE II score, mean (SD)	27.0 (7.3)	22.1 (7.2)	1.09 (1.08-1.11)	<.001	1.08 (1.07-1.10)	<.001
Bilirubin at randomization, mean (SD), mg/dL	2.0 (2.3)	1.5 (1.9)	1.01 (1.00-1.01)	<.001	1.01 (1.00-1.01)	<.001
Source of admission						
Accident and emergency department	246/701 (35)	660/2460 (27)	1 [Reference]	<.001	1 [Reference]	.57
Hospital floor (ie, wards)	171/701 (24)	560/2460 (23)	0.82 (0.65-1.03)		0.79 (0.61-1.02)	
Transfer from another ICU	34/701 (5)	99/2460 (4)	0.92 (0.60-1.38)		1.00 (0.63-1.57)	
Transfer from another hospital (except from another ICU)	76/701 (11)	233/2460 (10)	0.88 (0.65-1.17)		0.97 (0.70-1.35)	
Admitted following emergency surgery	154/701 (22)	789/2460 (32)	0.52 (0.42-0.66)		0.77 (0.28-1.90)	
Admitted following elective surgery	20/701 (3)	119/2460 (5)	0.45 (0.27-0.72)		0.78 (0.26-2.20)	
Admission type						
Nonoperative	532/701 (76)	1567/2460 (64)	1 [Reference]	<.001	1 [Reference]	.62
Operative	169/701 (24)	893/2460 (36)	0.56 (0.46-0.67)		0.78 (0.32-2.10)	
Primary site of infection						
Pulmonary	232/698 (33)	898/2456 (37)	1 [Reference]	<.001	1 [Reference]	.21
Intra-abdominal	121/698 (17)	562/2456 (23)	0.83 (0.65-1.06)		1.14 (0.85-1.52)	
Blood	147/698 (21)	381/2456 (16)	1.49 (1.17-1.89)		1.31 (1.00-1.71)	
Urinary	140/698 (20)	452/2456 (18)	1.20 (0.94-1.52)		1.34 (1.00-1.77)	
Other	58/698 (8)	163/2456 (7)	1.38 (0.98-1.91)		1.18 (0.80-1.71)	
First organism						
* Staphylococcus*	40/698 (6)	129/2455 (5)	1 [Reference]	.09	NA	NA
Other gram positives	146/698 (21)	502/2455 (20)	0.94 (0.63-1.41)		NA	
Gram negatives	159/698 (23)	469/2455 (19)	1.09 (0.74-1.64)		NA	
Fungi	40/698 (6)	120/2455 (5)	1.08 (0.65-1.78)		NA	
Other (including none identified)	313/698 (45)	1235/2455 (50)	0.82 (0.57-1.20)		NA	
Bacteremia						
Absence	559/701 (80)	2158/2458 (88)	1 [Reference]	<.001	1 [Reference]	<.001
Presence	142/701 (20)	300/2458 (12)	1.83 (1.46-2.27)		1.89 (1.47-2.41)	
Use of nephrotoxic antibiotics at randomization						
No	376/701 (54)	1454/2460 (59)	1 [Reference]	.01	1 [Reference]	.86
Yes	325/701 (46)	1006/2460 (41)	1.25 (1.06-1.48)		1.02 (0.84-1.23)	
Last MAP	71.8 (8.9)	72.7 (8.0)	0.99 (0.98-1.00)	.01	0.99 (0.98-1.01)	.33
NEE, µg/kg/min	0.33 (0.29)	0.22 (0.24)	1.16 (1.13-1.20)^b^	<.001	1.09 (1.05-1.14)^b^	<.001
MAP/NEE ratio, mm Hg/µg/kg/min	538.4 (901.9)	820.1 (1137.5)	0.96 (0.95-0.97)^c^	<.001	0.99 (0.97-1.00)^c^	.02
Use of vasopressin						
No	529/701 (75)	2165/2460 (88)	1 [Reference]	<.001	1 [Reference]	.003
Yes	172/701 (25)	295/2460 (12)	2.39 (1.93-2.94)		1.46 (1.14-1.87)	
Use of dobutamine, milrinone, or levosimendan						
No	664/701 (95)	2384/2460 (97)	1 [Reference]	.008	1 [Reference]	.24
Yes	37/701 (5)	76/2460 (3)	1.75 (1.16-2.59)		1.32 (0.83-2.06)	
Time from the onset of shock to randomization	22.9 (110.7)	19.6 (86.5)	1.00 (1.00-1.00)	.44	1.00 (1.00-1.00)	.53
Steroid received prior to randomization						
No	574/646 (89)	2064/2264 (91)	1 [Reference]	.08	NA	NA
Yes	72/646 (11)	200/2264 (10)	1.29 (0.97-1.71)		NA	
Treatment allocation						
Placebo	372/701 (53)	1200/2460 (49)	1 [Reference]	.05	1 [Reference]	.01
Hydrocortisone	329/701 (47)	1260/2460 (51)	0.84 (0.71-1.00)		0.79 (0.66-0.95)	

Abbreviations: APACHE, Acute Physiology and Chronic Health Evaluation; ICU, intensive care unit; KRT, kidney replacement therapy; MAP, mean arterial pressure; NEE, norepinephrine equivalent.

SI conversion factor: To convert bilirubin to micromoles per liter, multiply by 17.104.

^a^ Multivariate model includes treatment, age, sex, weight, APACHE II score, primary site of infection, NEE, MAP:NEE ratio, time from the onset of shock to randomization, and significant variables (*P* < .05) from univariate models.

^b^ Expressed for an increase of 0.1 unit.

^c^ Expressed for an increase of 100 units.

### Hemodynamic Markers of Risk for KRT Requirement

MAP at randomization was not associated with the development of a KRT requirement (OR, 0.99 [95% CI 0.98-1.01]; *P* = .33). The NEE dose was associated with KRT requirement (OR per 0.1-μg/kg/min increase, 1.09 [95% CI, 1.05 to 1.14]; *P* < .001). The MAP:NEE ratio at randomization was associated with the development of a KRT requirement (OR per 100–mm Hg/µg/kg/min increase, 0.99 [95% CI, 0.97 to 1.00]; *P* = .02). With respect to the postrandomization variables assessed (eTable 3 in Supplement 1), patients who required KRT had a longer time to shock resolution (mean [SD], 6.3 [6.8] days vs 3.7 [4.1] days; *P* < .001) and lower rates of enduring shock resolution (525 patients [75%] vs 2293 patients [94%]; *P* < .001).

### Source of Sepsis and KRT Requirement

The presence of bacteremia was significantly associated with the development of a KRT requirement (OR, 1.89 [95% CI, 1.47-2.41]; *P* < .001) (Table 3). The use of nephrotoxic antimicrobials was not significantly associated with the development of a KRT requirement (OR, 1.02 [95% CI, 0.84-1.23]; *P* = .86).

### Outcomes Following Commencement of KRT

**Figure 2.**
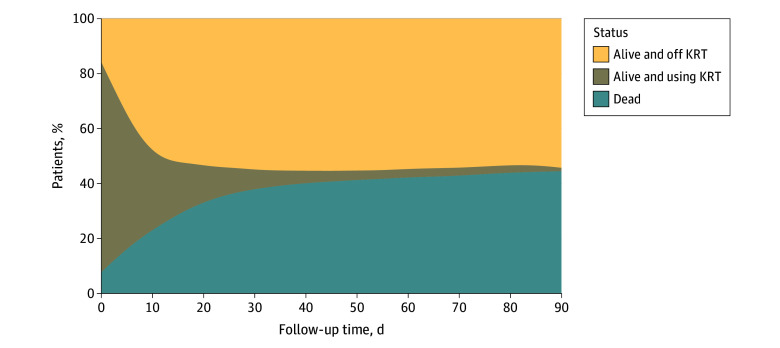
Outcomes for Patients With Kidney Replacement Therapy (KRT) Requirement By approximately day 30, few patients were weaned off KRT and remained alive.

At 90 days, 312 patients (44.7%) who required KRT had died, significantly higher than among patients who did not require KRT (486 patients [19.9%]) (*P* < .001). Patients who required KRT had fewer days alive and out of hospital at 90 days (mean [SD] 25.8 [29.3] days vs 46.0 [31.3] days; *P* < .001). There were 32 patients (4.6%) who continued dialysis treatment after hospital discharge (Figure 2). Of patients who developed a KRT requirement and survived, 364 (94.3%) had been liberated from dialysis at 90 days.

At 6 months, patients who required KRT had reduced survival compared with those who did not (mortality: 328 patients [72%] vs 561 patients [23%]; *P* < .001) and worse long-term quality of life scores (eTable 1 in Supplement 1). The mean (SD) 6-month EQ-5D-5L utility score was 0.3 (0.4) among patients requiring acute KRT, compared with 0.5 (0.4) among those who did not (*P* < .001).

### Associations With KRT Recovery

Source of admission, admission type (operative vs nonoperative), identified organisms, and primary site of infection were not associated with a significant difference in the number of days alive and free of KRT among patients who developed a KRT requirement (eTable 4 in Supplement 1). Older age was associated with reduced days alive and free of KRT (mean difference [MD] per 1-year increase, −0.54 [95% CI, −0.74 to −0.34] days; *P* < .001), while increasing weight was associated with an improved number of days alive and free of KRT (MD per 1-kg increase, 0.19 [95% CI, 0.09 to 0.30]; *P* < .001). Concomitant liver dysfunction (as reflected in presenting bilirubin) was associated with reduced days alive and free of KRT (MD, −0.10 [95% CI, −0.18 to −0.04]; *P* = .004).

Among patients who required KRT, a higher MAP at study randomization was associated with an increased number of days alive and free of KRT (MD per 1–mm Hg increase, 0.49 [95% CI, 0.17 to 0.82] days; *P* = .003). A higher vasopressor dose (NEE) at randomization was associated with a reduced number of days alive and free of KRT (MD per 0.1–μg/kg/min increase, −2.10 [95% CI, −3.16 to −1.05] days; *P* < .001). The presenting MAP:NEE ratio was not associated with the number of days alive and free of KRT. For patients who initiated KRT, randomization to receive hydrocortisone was not associated with a reduction in the number of days alive and free of KRT (MD, 1.28 [95% CI −4.31 to 6.87] days; *P* = .65).

## Discussion

In this large cohort study of patients with septic shock, nearly one-quarter of participants developed a new requirement for KRT. There was a high rate of both short- and long-term mortality and a reduced quality of life among patients who developed a KRT requirement. Only a small proportion of these patients survived with an ongoing KRT requirement. Treatment with hydrocortisone was associated with a reduced KRT requirement, robust to adjustment for confounding. For patients who did develop a subsequent KRT requirement, intravenous hydrocortisone use was not associated with a reduction in days alive and free of KRT.

In contrast to this cohort, neither the parent ADRENAL study^7^ nor other large RCTs of corticosteroids in septic shock^9^ have demonstrated differences in use of KRT associated with the use of hydrocortisone. This difference may relate to the smaller population sizes of previous studies,^10,11^ to the significant numbers of study participants included in several of these studies with established severe AKI at the time of study enrollment,^9,18^ or the high severity of illness (and attendant AKI risk) required for inclusion in the ADRENAL trial.

This post hoc analysis of a large RCT offers new insights. While post hoc assessments of secondary outcomes need to be interpreted with caution, there is biological plausibility to these findings relating to the impact of hydrocortisone, given recent large meta-analyses reporting a trend toward reduced organ dysfunction in association with steroids in septic shock,^19^ specific plausible cellular mechanisms of steroid effect described in animal models,^20,21,22^ and contemporary pathophysiological models that emphasize the role of renal cellular dysfunction and damage from dysregulated inflammatory responses.^5^ On the other hand, the absence of a significant association between days alive and free of KRT and hydrocortisone among patients who initiated dialysis and the short time between randomization and the commencement of KRT may suggest this to be a chance finding.

Previous explorations of the association between the recently proposed MAP:NEE ratio^14^ and subsequent KRT requirement have been limited to a single retrospective assessment of a mixed cohort (24% of whom had sepsis), and with an overall low KRT requirement (5.2%).^23^ After propensity matching, Liu et al^23^ reported findings similar to those described in this cohort of patients with sepsis. Our results suggest that shock severity, as reflected in the degree dependence of vasopressor (reflected in both the NEE dose and MAP:NEE ratio), is associated with the subsequent risk of severe SA-AKI, despite the careful maintenance of MAP within the target range (mostly 67-76 mm Hg in this cohort). While the clinical utility of the MAP:NEE ratio, with its modest statistical association with KRT requirement, may be limited, there may be a role for this measure to identify subphenotypes of septic shock for further testing of specific preventive interventions for SA-AKI, such as novel vasopressor types^24,25^ or adjunctive steroids.

### Strengths and Limitations

There are a number of notable strengths and limitations to this study. This is a large cohort of patients with septic shock, capturing a broad range of sources of sepsis and clinical contexts worldwide. The data were collected prospectively, and collection was independently monitored. By excluding patients with an established KRT requirement at baseline, the temporal association between the exposures of interest and outcome has been preserved. There is potential that this may have introduced some degree of selection bias. The dependence on KRT use as a surrogate for SA-AKI is a significant limitation. Although KRT requirement is an important patient-centered outcome, less severe (non–dialysis-requiring) AKI is not accounted for, and there may be instances where KRT was initiated for alternative indications than severe AKI. KRT initiation was not based on objective criteria. The assessment of the association between measures of hemodynamic compromise and KRT requirement is limited by the measurement of MAP and vasopressor requirement at a single time point at enrollment; patient fluid balance and volumes of fluid received were not included in the ADRENAL dataset. Additionally, the described associations of the included postrandomization variables (time to shock resolution and recurrence of shock) with KRT requirement in our cohort should be interpreted with caution.

## Conclusions

In this post hoc cohort study of patients with severe septic shock, the use of adjunctive hydrocortisone was associated with a reduced need for new onset KRT. New KRT requirement was associated with severe morbidity and mortality.

## Notes

Supplement 1. **eAppendix.** Calculation of Norepinephrine Equivalent (NEE) **eTable 1.** Outcomes for Those Requiring KRT **eTable 2.** Sensitivity Analyses **eTable 3.** Other Outcomes Associated With KRT Status **eTable 4.** Associations With Days Alive and Free of KRT: Univariate and Multivariate Models
eReferences.

Supplement 2.
Data Sharing Statement
