# Supplementary material for: Hydrocortisone and Risk Factors for Kidney Replacement Therapy in Septic Shock
Source: JAMA Netw Open. 2025 May 27;8(5):e2512279. doi: 10.1001/jamanetworkopen.2025.12279 (PMC12117457; doi:10.1001/jamanetworkopen.2025.12279)
Supplement: Supplement 1. — eAppendix. Calculation of Norepinephrine Equivalent (NEE) eTable 1. Outcomes for Those Requiring KRT eTable 2. Sensitivity Analyses eTable 3. Other Outcomes Associated With KRT Status eTable 4. Associations With Days Alive and Free of KRT: Univariate and Multivariate Models eReferences. [file jamanetwopen-e2512279-s001.pdf]

Supplemental Online Content

Donaldson LH, Devaux A, White KC, et al. Hydrocortisone and risk factors for kidney replacement therapy in septic shock. *JAMA Netw Open*. 2025;8(5):e2512279. doi:10.1001/jamanetworkopen.2025.12279

eAppendix. Calculation of Norepinephrine Equivalent (NEE)
eTable 1. Outcomes for Those Requiring KRT
eTable 2. Sensitivity Analyses
eTable 3. Other Outcomes Associated With KRT Status
eTable 4. Associations With Days Alive and Free of KRT: Univariate and Multivariate Models
eReference.

This supplemental material has been provided by the authors to give readers additional information about their work.

© 2025 Donaldson LH et al. JAMA Network Open.

eAppendix. Calculation of Norepinephrine Equivalent (NEE)

NEE = **Noradrenaline** dose (mcg/kg/min) + **Adrenaline** dose (mcg/kg/min) + 1/100 × **dopamine** dose (mcg/kg/min) + 0.1 if noted to be on **vasopressin**

Note that the dose of vasopressin was not recorded in the ADRENAL study. The above adjustment was based on the typical starting dose of vasopressin 0.04u/min, and a conversion factor of 2.5. (1)

eTable 1: Outcomes for those requiring KRT

**Variables**	**KRT**<br>N = 701	**No KRT**<br>N = 2,460	**p-value**
**Days alive and out of the ICU, mean (SD)**	40.6 (36.3)	65.1 (31.3)	<0.001
**Days alive and out of the hospital, mean (SD)**	25.8 (29.2)	46.0 (31.3)	<0.001
**90-day mortality**	312 / 698 (44.7%)	486 / 2442 (19.9%)	<0.001
**6-month mortality**	328 / 695 (47.2%)	561 / 2407 (23.3%)	<0.001
**Hospital Readmission Rates**	38 / 701 (5.4%)	201 / 2460 (8.2%)	0.02
**Quality of life scores**			
**6-month QoL EQ5D5L Utility score (mean, SD)[1]**	0.3 (0.4)	0.5 (0.4)	<0.001
**QoL Utility scores with deceased patients excluded (sensitivity analysis)[2]**	0.6 (0.4)	0.7 (0.3)	0.36
**ED5D5L Visual Analogue Scale (mean, SD)[3]**	70.9 (20.4)	71.2 (20.9)	0.78

1) Deceased patients coded '0', for this primary analysis.
2) Sensitivity analysis which includes only survivors.
3) Only survivors included

eTable 2: Sensitivity Analyses

**Outcomes**	**Treatment allocation**			
	**Hydrocortisone**	**Placebo**	****OR/MD (95% CI)****	**p-value**
**Adjusted analysis for other predictors of KRT requirement [1]**				
**Use of KRT[1]**	329 / 1,589 (20.7%)	372 / 1,572 (23.7%)	OR 0.81 [0.68 - 0.97]	0.02
**Number of days alive and free for KRT[1]**	69.6 (34.0)	68.1 (35.0)	MD 1.60 [-0.73 - 3.93]	0.18
**Analysis excluding patients who commenced KRT <1 day after randomisation (as well as KRT at baseline and/or chronic dialysis requirement)[2]**				
**Use of KRT**	189 / 1,449 (13.0%)	219 / 1,419 (15.4%)	OR 0.82 [0.66 – 1.02]	0.08
**Analysis excluding patients who commenced KRT <2 days after randomisation (as well as KRT at baseline and/or chronic dialysis requirement)[2]**				
**Use of KRT**	114 / 1,374 (8.3%)	144 / 1,344 (10.7%)	OR 0.78 [0.59 – 1.01]	0.06

1. adjusted for age, sex, APACHE II score, bilirubin at enrolment, primary site of infection, time from the onset of shock to randomization, use of vasopressin and last MAP at enrolment as fixed effects.

2. adjusted for admission type as a fixed effect, and sites as a random effect, only.

© 2025 Donaldson LH et al. JAMA Network Open.

eTable 3: Other Outcomes associated with KRT Status

Variables	KRT<br>N = 701	No KRT<br>N = 2,460	p-value
**Time to shock resolution (days, mean (SD)**	6.3 (6.8)	3.7 (4.1)	**<0.001**
**Persistent shock resolution (N, %)**	525 / 701 (75%)	2,293 / 2452 (94%)	**<0.001**

eTable 4: Associations with days alive and free of KRT - univariate and multivariate models

	**Univariate**		**Multivariate**	
**Variables**	**MD (95% CI)**	**p-value**	**MD (95% CI)**	**p-value**
**Age at randomisation (years)**	-0.54 (-0.73, -0.34)	<0.001	-0.52 (-0.72, -0.31)	<0.001
**Sex**		0.33		0.22
Female	—		—	
Male	-2.90 (-8.74, 2.94)		-3.64 (-9.49, 2.21)	
**Weight (in kg)**	0.22 (0.12, 0.32)	<0.001	0.19 (0.08, 0.29)	<0.001
**APACHE II Score**	-0.37 (-0.75, 0.02)	0.06	-0.06 (-0.47, 0.35)	0.78
**High bilirubin**	-0.08 (-0.15, -0.01)	0.03	-0.11 (-0.18, -0.04)	0.003
**Source of admission**		0.05		0.75
Accident and Emergency Department	—		—	
Hospital Floor (i.e. wards)	-9.19 (-16.64, -1.74)		-3.16 (-10.66, 4.34)	
Transfer from another ICU	6.10 (-7.55, 19.75)		1.94 (-11.51, 15.38)	
Transfer from another hospital (except from another ICU)	2.53 (-7.26, 12.33)		3.47 (-6.04, 12.97)	
Admitted from Theatre following EMERGENCY surgery	-6.84 (-14.51, 0.84)		-3.10 (-11.58, 5.38)	
Admitted from Theatre following ELECTIVE surgery	-7.59 (-24.94, 9.75)		3.75 (-13.60, 21.09)	
**Admission type**		0.06		
Non-operative	—			
Operative	-6.26 (-12.87, 0.34)			
**Primary site of infection**		0.07		0.11
Pulmonary	—		—	
Intra abdominal	-5.44 (-13.83, 2.94)		-2.41 (-11.22, 6.39)	
Blood	-3.41 (-11.32, 4.51)		-2.52 (-10.25, 5.21)	
Urinary	0.20 (-7.81, 8.20)		-0.05 (-8.56, 8.46)	
Other	11.42 (0.45, 22.39)		12.80 (1.79, 23.82)	
**First organism**		0.31		
Staphylococcus	—			
Other gram positives	7.09 (-6.28, 20.46)			
Gram Negatives	4.91 (-8.36, 18.18)			
Fungi	-6.93 (-23.68, 9.83)			
Other (incl nil identified)	3.53 (-9.05, 16.11)			
**Bacteraemia**		0.16		
Absence	—			
Presence	5.00 (-2.05, 12.06)			
**Use of nephrotoxic antibiotics at randomization**		0.08		
No	—			

© 2025 Donaldson LH et al. JAMA Network Open.

	Univariate		Multivariate	
Variables	MD (95% CI)	p-value	MD (95% CI)	p-value
Yes	5.15 (-0.53, 10.82)			
**Last MAP**	0.51 (0.19, 0.83)	0.002	0.49 (0.17, 0.82)	0.003
**NEE**	-2.08 (-3.04, -1.11)[1]	<0.001	-2.09 (-3.15, -1.03)[1]	<0.001
**MAP/NEE ratio**	0.19 (-0.12, 0.51)[2]	0.23	-0.23 (-0.57, 0.10)[2]	0.16
**Use of Vasopressin**		0.51		
No	—			
Yes	2.20 (-4.39, 8.80)			
**Use of Dobumatine, Milrinone or Levosimendan**		0.39		
No	—			
Yes	-5.58 (-18.24, 7.07)			
**Time from the onset of shock to randomisation**	-0.01 (-0.04, 0.01)	0.32	-0.02 (-0.04, 0.01)	0.21
**Steroid received**		0.13		
No	—			
Yes	7.26 (-2.08, 16.59)			
**Treatment allocation**		0.75		0.65
Placebo	—		—	
Hydrocortisone	0.92 (-4.77, 6.60)		1.28-4.31, 6.87)	

1. Expressed for an increase of 0.1 unit; 2. Expressed for an increase of 100 units
Multivariate model includes treatment, age, sex, weight, APACHE II score, primary site of infection, NEE, MAP/NEE, time from the onset of shock to randomisation and significant variables (p<0.05) from univariate mode

Reference

1. Kotani Y, Di Gioia A, Landoni G, Belletti A, Khanna AK. An updated “norepinephrine equivalent” score in intensive care as a marker of shock severity. Critical Care. 2023;27(1):29.

© 2025 Donaldson LH et al. JAMA Network Open.
