# Supplementary material for: Hydrocortisone and Risk Factors for Kidney Replacement Therapy in Septic Shock
Source: JAMA Netw Open. 2025 May 27;8(5):e2512279. doi: 10.1001/jamanetworkopen.2025.12279 (PMC12117457; doi:10.1001/jamanetworkopen.2025.12279)
Supplement: Supplement 2. — Data Sharing Statement [file jamanetwopen-e2512279-s002.pdf]

Data Sharing Statement

Donaldson. Hydrocortisone and Risk Factors for Kidney Replacement Therapy in Septic Shock. *JAMA Netw Open*. Published May 27, 2025. doi:10.1001/jamanetworkopen.2025.12279

Data
Data available: No

Additional Information
Explanation for why data not available: Data sharing is subject to The George Institute for Global Health data sharing policy available online (https://www.georgeinstitute.org.au/data-sharing-policy)
